# Accumulation of Target Gene Mutations Confers Multiple Resistance to ALS, ACCase, and EPSPS Inhibitors in *Lolium* Species in Chile

**DOI:** 10.3389/fpls.2020.553948

**Published:** 2020-10-28

**Authors:** José G. Vázquez-García, Ricardo Alcántara-de la Cruz, Candelario Palma-Bautista, Antonia M. Rojano-Delgado, Hugo E. Cruz-Hipólito, Joel Torra, Francisco Barro, Rafael De Prado

**Affiliations:** ^1^Department of Agricultural Chemistry and Edaphology, University of Córdoba, Córdoba, Spain; ^2^Departamento de Química, Universidade Federal de São Carlos, São Carlos, Brazil; ^3^Department d’Hortofruticultura, Botànica i Jardineria, Agrotecnio, Universitat de Lleida, Lleida, Spain; ^4^Department of Plant Breeding, Institute for Sustainable Agriculture, CSIC (IAS-CSIC), Córdoba, Spain

**Keywords:** glyphosate, diclofop-methyl, iodosulfuron methyl-sodium, italian ryegrass, perennial ryegrass, rigid ryegrass

## Abstract

Different *Lolium* species, common weeds in cereal fields and fruit orchards in Chile, were reported showing isolated resistance to the acetyl CoA carboxylase (ACCase), acetolactate synthase (ALS) and 5-enolpyruvylshikimate-3-phosphate synthase (EPSPS) inhibiting herbicides in the late 1990s. The first case of multiple resistance to these herbicides was *Lolium multiflorum* found in spring barley in 2007. We hypothesized that other *Lolium* species may have evolved multiple resistance. In this study, we characterized the multiple resistance to glyphosate, diclofop-methyl and iodosulfuron-methyl-sodium in *Lolium rigidum*, *Lolium perenne* and *Lolium multiflorum* resistant (R) populations from Chile collected in cereal fields. *Lolium* spp. populations were confirmed by AFLP analysis to be *L. rigidum*, *L. perenne* and *L. multiflorum*. Dose-response assays confirmed multiple resistance to glyphosate, diclofop-methyl and iodosulfuron methyl-sodium in the three species. Enzyme activity assays (ACCase, ALS and EPSPS) suggested that the multiple resistance of the three *Lolium* spp. was caused by target site mechanisms, except the resistance to iodosulfuron in the R *L. perenne* population. The target site genes sequencing revealed that the R *L. multiflorum* population presented the Pro-106-Ser/Ala (EPSPS), Ile-2041-Asn++Asp-2078-Gly (ACCase), and Trp-574-Leu (ALS) mutations; and the R *L. rigidum* population had the Pro-106-Ser (EPSPS), Ile-1781-Leu+Asp-2078-Gly (ACCase) and Pro-197-Ser/Gln+Trp-574-Leu (ALS) mutations. Alternatively, the R *L. perenne* population showed only the Asp-2078-Gly (ACCase) mutation, while glyphosate resistance could be due to *EPSPS* gene amplification (no mutations but high basal enzyme activity), whereas iodosulfuron resistance presumably could involve non-target site resistance (NTSR) mechanisms. These results support that the accumulation of target site mutations confers multiple resistance to the ACCase, ALS and EPSPS inhibitors in *L. multiflorum* and *L. rigidum* from Chile, while in *L. perenne*, both target and NTSR could be present. Multiple resistance to three herbicide groups in three different species of the genus *Lolium* in South America represents a significant management challenge.

## Introduction

*Lolium* grasses are problematic weeds around the world (Salas et al., 2012; Menegat et al., 2016), causing yield losses in small grains, orchards, and vineyards, as well as in non-agricultural lands (Travlos et al., 2018). There are 17 *Lolium* species described, but the most problematic ones in agricultural producing systems are *L. multiflorum* (Lam.) Husnot (Italian ryegrass), *L. perenne* L. (perennial ryegrass) and *L. rigidum* Gaudin (rigid ryegrass) (Menegat et al., 2016). The last species is considered the most economically important weed in Australia, while *L. multiflorum* and *L. perenne* are frequents weeds in crops of North and South America and the Mediterranean region (Heap, 2020).

Over the past three decades, control of *Lolium* grasses has been carried out using almost exclusively PRE- and POST-emergence herbicides, exerting high selection pressure that has resulted in resistance to most herbicide sites of action (SoAs) (Tehranchian et al., 2018). The first case of resistance in *L. rigidum* was found in canola, spring barley, and wheat fields in Australia in 1982. Since then, herbicide resistance cases in *Lolium* species have increased substantially over time (Heap, 2020). There are currently 67, 9, and 48 cases of herbicide resistance in *L. multiflorum*, *L. perenne*, and *L. rigidum*, respectively, recorded in the database of the *International Survey of Herbicide Resistant Weeds*. At least half of these are cases of cross and/or multiple resistance (Heap, 2020).

*Lolium* grasses are difficult to control chemically because they have the capacity to evolve different herbicide resistance mechanisms very quickly, depending on the distribution of resistant individuals within the cultivated areas, seed viability, obligated cross-pollination, genetic variability and high phenotypic plasticity (Travlos et al., 2018). For example, *L. rigidum* has evolved resistance to 14 different herbicide SoAs, and *L. multiflorum* has evolved resistant to 9 SoAs (Heap, 2020). In addition, because *Lolium* weed species generally are diploids (Dalton et al., 1999; Busi and Powles, 2013), they are capable of hybridizing with each other, resulting in populations with homozygous and heterozygous individuals that can carry multiple resistance alleles (Menegat et al., 2016). In the long term, this situation reduces the options for diversification of alternative herbicides for the integrated management of these weeds (Fernández et al., 2017). In addition, resistance alleles are generally not linked and accumulate independently, i.e., *Lolium* species are able to accumulate different resistance mechanisms, so different combinations of resistance alleles can be found resulting in complex multiple herbicide resistance profiles (Tan et al., 2007; Martins et al., 2014). The most serious multiple herbicide resistance cases are those including resistance to glyphosate (5-enolpyruvylshikimate-3-phosphate synthase [EPSPS] inhibitor) (Fernández-Moreno et al., 2017a). Populations of *L. multiflorum* showing resistance to glyphosate, sethoxydim (acetyl CoA carboxylase [ACCase] inhibitor), and paraquat have been found in the United States (Tehranchian et al., 2018); *L. rigidum* populations resistant to glyphosate, paraquat, ACCase and acetolactate synthase (ALS) inhibitors have been found in Australia (Yu et al., 2007, 2008). Additionally, different populations of *Lolium* species with multiple resistance to up to four herbicide SoAs have been reported throughout the Mediterranean region in Europe (Collavo and Sattin, 2014; Fernández et al., 2017; Travlos et al., 2018).

In Chile, herbicide resistance is not recent, and of the 19 known cases, nine involve *Lolium* species with single and/or multiple resistance occurring in small grain fields (barley, canola, cereals, lupins, spring and winter wheat) and fruit orchards (Heap, 2020). In Chile, *L. rigidum*, *L. multiflorum* and *L. perenne* were found with resistance to ACCase inhibitors in small grains fields in 1997, 1998 and 2001, respectively (Heap, 2020). *Lolium multiflorum* was the first species to show resistance to glyphosate, and it was found in fruit orchards in 2001 (Perez and Kogan, 2003). The first case of resistance to ALS inhibitors was also *L. multiflorum*, found in wheat fields in 2002, and this case was the first one of multiple resistance, since this species also showed resistance to glyphosate (Heap, 2020). Because ACCase- and ALS-inhibiting herbicides became recurring alternatives to manage glyphosate resistance and vice versa, we hypothesized that populations of *Lolium* species from Chile may have had multiple resistance to these three herbicide groups, since they can select herbicide resistance in few generations, even when exposed to subdoses (Busi et al., 2012). However, the resistance mechanisms were characterized only for *L. multiflorum* resistance to glyphosate (Perez and Kogan, 2003; Michitte et al., 2007). In addition, herbicide resistance in *Lolium* species is widely dispersed in neighboring Latin American countries (Heap, 2020), and only in Brazil is it estimated that *L. multiflorum* affects more than 4.2 million ha (Alcántara-de la Cruz et al., 2020). However, with the exception of the recent characterization of the resistance mechanisms to ACCase inhibitors in *L. perenne* from Argentina (Yanniccari and Gigón, 2020), there are no studies of resistance mechanisms in *Lolium* species from South America (*L. multiflorum* and *L. perenne*) other than for glyphosate (Yanniccari et al., 2017; Barroso et al., 2018).

In this work, the multiple resistances to glyphosate, diclofop-methyl and iodosulfuron-methyl-sodium (inhibiting herbicides of EPSPS, ACCase and ALS enzymes, respectively) were characterized in three putative resistant (R) populations of *L. rigidum*, *L. perenne* and *L. multiflorum*, collected in spring barley and winter wheat fields in the Regions VIII (San Bernardo and Olivar) and IX (Vilcún) in Chile. The possible target-site resistance (TSR) mechanisms involved were studied in these three populations, being a one-of-a-kind study for South America.

## Materials and Methods

### Chemicals

Trade formulations of glyphosate (Roundup 480, Monsanto Europe), diclofop-methyl (Firelo, Dupont Spain) and iodosulfuron-methyl-sodium (Hussar, Bayer CropScience Spain) were used for the dose-response assays. Glyphosate, diclofop-acid and iodosulfuron of analytical grade (Sigma-Aldrich, Spain) with a purity of 99% were used for enzymatic assays.

### Plant Material

Seeds of the different R *Lolium* spp. (*L. rigidum*, *L. perenne*, and *L. multiflorum*) were supplied in 2010 by Dr. Nelson Espinoza (INIA of Carillanca, Chile). Mature seeds were collected from different fields of spring barley (Region VIII) and winter wheat (Region IX) grown under direct drilling systems for several years (Espinoza et al., 2009). Spring weeds were removed in pre-sowing with glyphosate, and during the crop season, ALS- (iodosulfuron) and/or ACCase- (diclofop, haloxyfop, and others) inhibiting herbicides were used for weed control. A screening test was conducted on the R populations to eliminate susceptible individuals from the field-collected seed, which consisted of germinating the seeds of the putative R populations and treating the seedlings at field doses with diclofop-methyl (900 g ai ha^–1^), glyphosate (720 g ae ha^–1^) and iodosulfuron (5 g ai ha^–1^). Individuals surviving these applications were allowed to grow to maturity to collect the purified seeds used in this study. Seeds of populations of each *Lolium* species that were susceptible (S) to these herbicides were collected in areas near these crops.

Seeds of the R and S *Lolium* spp. populations were placed for germination in 9-cm Petri dishes containing moistened filter paper. Petri dishes were placed in a growth chamber at 26/18°C (day/night), with relative humidity of 60% and a photoperiod of 16 h at a light density of 850 mmol m^–2^ s^–1^. Seedlings were transplanted into plastic pots in 250 mL substrate (sand and peat 1:1). Pots were returned to the growth chamber, and the plants were irrigated as necessary until ready for use (plants having 3–4 true leaves).

### Molecular Characterisation of *Lolium* Species

One population of each *Lolium* species, i.e., *L. multiflorum*, *L. rigidum* (harvested in southern Spain) and *L. perenne* (North of Portugal) were included as reference populations in the molecular analysis with amplified fragment length polymorphism (AFLP) markers. The molecular identity of the reference populations has been previously confirmed. Twelve plants of each *Lolium* spp. population were used for DNA extraction from leaf tissue (50 mg plant^–1^) by using the Speedtool DNA Extraction Plant kit (Biotools, Madrid, Spain). DNA was quantified in a NanoDrop ND-1000 spectrophotometer. AFLP analysis was carried out using the fluorescent AFLP IRDye kit for Large Plant Genome Analysis (LI-COR Biosciences) following the manufacturer’s instructions. Twelve primer pairs [E36-M48 (E-ACC MCAC)/E36-M60 (E-ACC MCTC)/E37-M49 (E-ACG MCAG)/E38-M50 (E-ACT MCAT)/E40-M61 (E-AGC MCTG)/E35-M49 (E-ACA MCAG)/E36-M49 (E-ACC MCAG)/E35-M61 (E-ACA MCTG)/E40-M62 (E-AGC MCTT)/E32-M60 (E-AAC MCTC)/E33-M50 (E-AAG MCAT)/E35-M48 (E-ACA MCAC)] were used for selective amplification (Fernández et al., 2017; Fernández-Moreno et al., 2017a,b). AFLP products were separated in a polyacrylamide electrophoresis using an automated sequencer (LICOR 4300). Polymorphic AFLP markers (12) and primers were identified, and individuals were scored for presence (1) or absence (0) of AFLP fragments using the software package SAGAMX 2 GENERATION. Genetic distances were calculated using Jaccard’s coefficients of similarity. Grouping of the genotypes was determined by using the unweighted pair group method with arithmetic mean (UPGMA). The analysis was performed with AFLP marker data in the program NTSYSpc 2.2.

### Dose–Response Assays

R and S *Lolium* spp. plants were treated with different doses of diclofop (0, 100, 200, 400, 800, 1,600, 3,200, 4,000 g ai ha^–1^), glyphosate (0, 31.25, 62.5, 125, 250, 500, 1,000, 1,500, 2,000, 4,000 g ae ha^–1^) and iodosulfuron (0, 1, 2, 4, 8, 16, 32, 64, 128 g ai ha^–1^) in a laboratory chamber (SBS-060 De Vries Manufacturing, Hollandale, MN, United States) equipped with 8002 flat fan nozzles and delivering 200 L ha^–1^ at 250 KPa. The experiments were based on a completely random design with eight replications by dose; and experiments were repeated. Plant mortality and above-ground fresh weight were evaluated 28 days after treatment (DAT), and data were expressed as the percentage of the untreated control. Herbicide rates reducing the plant growth (GR_50_) or causing plant mortality by 50% (LD_50_) with respect to the untreated control were determined for each *Lolium* spp. population and herbicide.

### Enzyme Activity Assays

#### EPSPS Activity

The extraction and activity of the EPSPS enzyme was carried out following the methodology described by Dayan et al. (2015). Five grams of foliar tissue from the different *Lolium* spp. populations were ground with liquid N_2_ to a fine powder. Samples were transferred to 50 mL Falcon tubes containing 25 mL of extraction buffer (100 mM MOPS, 5 mM EDTA, 10% glycerol, 50 mM KCl, and 0.5 mM benzamidine) with 70 μL of ß-mercaptoethanol (10 mM) and 1% in polyvinylpolypyrrolidone (PVPP) to extract the EPSPS. Samples were vortexed for 5 min, avoiding foaming, and then centrifuged (18,000 *g*, 30 min, 4°C). Supernatant was filtered using a cheesecloth in a cold beaker and then slowly added (NH_4_)_2_SO_4_, was then slowly added while supernatant was under continuous stirring until a 45% solution of (NH_4_)_2_SO_4_ (w/v) was obtained. After addition of (NH_4_)_2_SO_4_ the sample was stirred for 30 min and then centrifuged (15,000 *g*, 30 min, 4°C). This step was repeated once more to obtain a 70% (NH_4_)_2_SO_4_ (w/v) solution to precipitate the fraction that contained the EPSPS activity. Supernatants were discarded, and pellets were resuspended in 1–2 mL assay buffer (100 mM MOPS, 1 mM MgCl_2_, 10% glycerol (v/v), 2 mM sodium molybdate, 200 mM NaF). Samples were dialysed using Slide-A-Lyzer dialysis cassettes (1000-MWC, Thermo Scientific, Meridian, IL, United States) overnight in 2 L of dialysis buffer (100 mM MOPS and 5 mM EDTA) at 4°C on a stir plate. The final pH of the buffers was adjusted to 7.0 with HCl or NaOH. The concentration of total protein soluble (TPS) was determined by Bradford assay (Bradford, 1976). Basal and enzyme EPSPS activities were determined in a continuous assay quantifying the inorganic phosphate (Pi) released from shikimate-3-phosphate with the EnzCheck phosphate assay kit (Invitrogen, Carlsbad, CA, United States) following the manufacturer’s instructions. The glyphosate concentrations used were: 0, 0.1, 1, 10, 100 and 1,000 μM. The amount Pi released was measured for 10 min at 360 nm in a spectrophotometer (DU-640, Beckman Coulter Inc. Fullerton, CA, United States). The EPSPS activity was calculated by determining the amount of Pi (μmol) released in μg of TSP^–1^ min^–1^. EPSPS enzyme activity was expressed as percentage of enzyme activity in presence of glyphosate with respect to the control (basal activity without glyphosate). The experiment was repeated for all populations, and each glyphosate concentration had three technical replicates.

#### ACCase Activity

The *in vitro* ACCase enzyme activity was performed following the protocol described by De Prado et al. (2005). Six grams of fresh weight were taken from new leaves of the different *Lolium* spp. populations for the extraction step. The samples were ground using liquid N_2_ and then added to 24 mL extraction buffer (0.1 M Hepes-KOH at pH 7.5, 0.5 M glycerol, 2 mM EDTA and 0.32 mM PMSF). After mixing for 3 min with a magnetic stirrer and then filtered using a cheesecloth, the samples were centrifuged (24,000 *g*, 30 min, 4°C). the supernatant was fractioned with (NH_4_)_2_SO_4_ and centrifuged (12,000 *g*, 10 min at 4°C). The pellets were resuspended in 1 mL S400 buffer (0.1 M Tricine-KOH at pH 8.3, 0.5 M glycerol, 0.05 M KCl, 2 mM EDTA and 0.5 mM DTT). The homogenate was applied to a desalting column (PD-10 columns, Sephadex G-25 M, Amersham Biosciences AB, SE-751 84, Uppsala, Sweden) and eluted in 2 mL of S400 buffer. The specific protein concentrations were determined by Bradford assay (Bradford, 1976). The enzyme activity was measured through the ATP-dependent incorporation of NaH[^14^C]O_3_ into [^14^C]malonyl-CoA. The reaction was conducted at 34°C in 7 mL scintillation vials with 0.1 M Tricine-KOH at pH 8.3, 0.5 M glycerol, 0.05 M KCl, 2 mM EDTA, 0.5 mM DTT, 1.5 mM MgCl_2_, 15 mM NaH[^14^C]O_3_ (1.22 MBq μmol^–1^), 50 μL of enzyme extract, 5 mM acetyl-CoA in a final volume of 0.2 mL. The reaction was stopped after 5 min by adding 30 μL of HCl 4N. After the drying step, 0.5 mL of ethanol-water solution (1:1, v/v) was added to the vial, followed by 5 mL of scintillation cocktail (Ultima Gold, Perkin-Elmer, BV BioScience Packard). Radioactivity was determined by liquid scintillation spectrometry. One unit of ACCase activity was defined as 1 μmol malonyl CoA formed min^–1^. The diclofop-acid concentrations were: 0, 0.1, 1, 10, 100, 1,000 and 10,000 μM. The experiment was repeated with three replicates for each herbicide concentration.

#### ALS Activity

ALS activity in presence of different iodosulfuron concentrations was determined *in vitro* following the protocol described by Rojano-Delgado et al. (2015). Three grams of young leaf tissue were powdered using liquid N_2_. Polyvinylpyrrolidone (0.5 g) was added to the fine powder as well as extraction buffer [1M K-phosphate buffer solution (pH 7.5), 10 mM sodium pyruvate, 5 mM MgCl_2_, 50 mM thiamine pyrophosphate, 100 μM flavin adenine dinucleotide, 12 mM dithiothreitol and glycerol–water (1:9, v/v)] in a proportion of 1:2 tissue–buffer. Samples were agitated for 10 min at 4°C, filtered through a cheesecloth, and centrifuged (20,000 *g* for 20 min). The supernatant was immediately used for the enzyme assays. For the ALS enzyme activity, 90 μL of enzyme extract was used with 110 μL of freshly prepared assay buffer [0.08 M K-phosphate buffer solution (pH 7.5), 0.5 M sodium pyruvate, 0.1 M MgCl_2_, 0.5 mM thiamine pyrophosphate and 1,000 μM flavin adenine dinucleotide]. The iodosulfuron concentrations assayed were: 0, 0.1, 1, 10, 50, 100, 500, 1,000 and 5,000 μM. Aliquots of 250 μL of a solution 0.04 M K_2_HPO_4_ at pH 7.0 were added. This mixture was incubated for 1 h at 37°C. Afterward, the reaction was stopped by adding 50 μL of H_2_SO_4_/water 1:10 (v/v). To decarboxylate acetolactate to acetoin, the tubes with the mixture were heated for 15 min at 60°C. A colored complex (λ 520 nm) formed after the addition of 250 μL of creatine (5 g L^–1^ freshly prepared in water) and 250 μL of 1-naphthol (50 g L^–1^ freshly prepared in 5 N NaOH) prior to incubation at 60°C for 15 min. Total protein content was determined by the Bradford method (Bradford, 1976), based on measurement of the absorbance at 595 nm of an acidic solution of Coomassie Brilliant Blue G-250 after binding to proteins. The experiment was repeated and with three replicates for each concentration of the herbicide.

### RNA Extraction and Target-Site Genes Sequencing

Samples (∼100 mg) of foliar tissue were taken from 20 seedlings from the R and S *Lolium* spp. populations. Then, both R plants were treated with glyphosate, diclofop and iodosulfuron at a rate of 720 (g ae ha^–1^), 400 and 5 g ai ha^–1^, respectively, as was done in the dose-response assays. Only R plants that survived these application rates 21 DAT were considered for molecular analyses (Cruz-Hipolito et al., 2015). Total RNA was isolated from leaves using TRIzol reagent (Invitrogen, Carlsbad, CA, United States) following the manufacturer’s instructions. RNA was treated with TURBO DNase (RNase-Free; Ambion, Warrington, United Kingdom) to eliminate any DNA contamination and stored at −80°C. First strand complementary DNA (cDNA) synthesis was carried out with 2 μg RNA per sample using an iScript cDNA Synthesis Kit (Bio-Rad Laboratories, Inc. CA, United States). The primers to amplify the ACCase (two fragments), ALS (two fragments), EPSPS genes and the PCR conditions are described in Table 1. Each PCR reaction was conducted in a total volume of 25 μL [50 ng of cDNA, 0.2 μM of each primer, 0.2 mM dNTP mix (PE Applied Biosystems; Life Technologies S.A., Spain), 2 mM MgCl2, 1X buffer, and 0.625 units of a 100:1 enzyme mixture of non-proofreading (*Thermus thermophilus*) and proofreading (*Pyrococcus furiosus*) polymerases (BIOTOOLS, Madrid, Spain)]. Each cDNA sample, i.e., cDNA of each plant, was amplified in triplicate. An aliquot of the PCR product (10 μL) was loaded in a 1% agarose gel to check the correct band amplification. The rest of the PCR product (15 μL) was purified using ExoSAP-IT^®^ for PCR Product Clean-Up (USB, Cleveland, OH, United States) as indicated by the manufacturers and sequenced. Sanger sequencing was carried out by the SCAI DNA sequencing service of the University of Córdoba, Spain. Gene sequences of the R and S *Lolium* species were verified and assembled using the Geneious 8.1.8 (Biomatters Ltd, Auckland, New Zealand) software, to identify mutations responsible for conferring herbicide resistance.

**TABLE 1:** Primers and PCR conditions used to amplify the 5-enolpiruvylshikimate-3-phosphate synthase (*EPSPS*), acetyl-coenzyme A carboxylase (*ACCase*, two fragments) and acetolactate synthase (*ALS*) genes to identify potential mutations responsible for herbicide resistance in *Lolium* species in Chile.

Target gene	Primers	Sequences 5′→3′	Fragment length	References	PCR conditions
*EPSPS*	Forward	AGCTGTAGTCGTTGGCTGTG	120 bp	Fernández-Moreno et al., 2017b	1 cycle at 94°C for 3 min; 35 cycles of 94°C for 30 s; 55°C for 30 s and 72°C for 60 s; and a final extension step of 10 min at 72°C.
	Reverse	GCCAAGAAATAGCTCGCACT			
*ACCase*	CP1-F	CAACTCTGGTGCTIGGATIGGCA	551 bp	Martins et al., 2014	1 cycle at 95°C for 30 s; 37 cycles of 95°C for 10 s; 60°C for 15 s and 72°C for 45 s; and a final extension step of 10 min at 72°C.
	CP1-R	GAACATAICTGAGCCACCTIAATATATT			
	ACCF2	ATCCTCGTGCAGCCATAAGTG	510 bp	Tehranchian et al., 2018	1 cycle at 95°C for 3 min; 40 cycles of 95°C for 30 s; 57°C for 45 s and 72°C for 60 s; and a final extension step of 5 min at 72°C.
	ACCR2	TGCATTCTTGGAGTTCCTCTG			
*ALS*	ALSF197	ACTCCATCCCCATGGTGGC	1,449 bp	Yu et al., 2008	1 cycle at 94°C for 4 min; 35 cycles of 94°C for 30 s; 55°C for 30 s and 72°C for 90 s; and a final extension step of 5 min at 72°C.
	ALSR653	TCCTGCCATCACCTTCCATG			

### Statistical Analysis

Dose-response and enzyme activity data were subjected to non-linear regression analysis using the log-logistic equation *Y* = *d*/1+(*x*/*g*)*^b^*); where *Y* represents the percentage of fresh weight reduction, plant survival and enzyme activity inhibition with respect to the control, *d is* the limits of the upper asymptote, *b* is the slope of the curve, *g* is the herbicide rate at the inflection point (i.e., GR_50_, LD_50_ or I_50_), and *x* (independent variable) is the herbicide dose. Using this equation, the GR_50_, LD_50_ and I_50_ (herbicide concentration to inhibit the ACCase, EPSPS and/or ALS activity by 50%) of each population of each species were calculated. Regression analyses were conducted using the ‘*drc*’ package with program R version 3.2.5 (Ritz et al., 2015). Resistance factors (RF = R/S) were calculated as the ratio of R to-S GR_50_, LD_50_ or I_50_.

Specific enzyme activity data were submitted to one-way ANOVA (for *P* < 0.05). The Tukey test was used for mean comparison (95% confidence level) to test for significant differences between populations within each species and for each enzyme.

## Results

### Molecular Characterisation of *Lolium* Species

**FIGURE 1:**
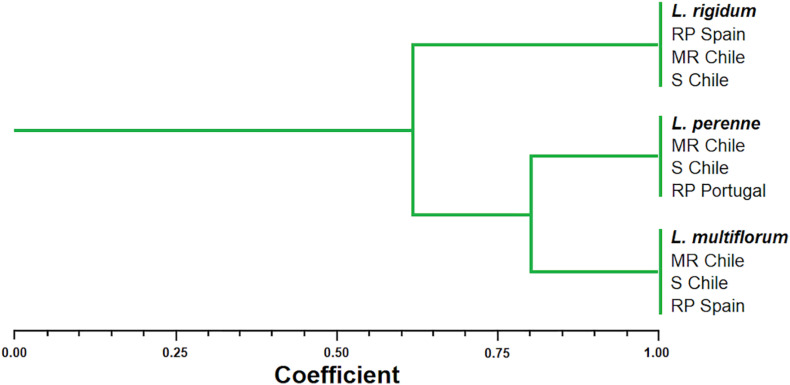
Dendrogram of the genetic similarities among multiple herbicide resistant (MR) and susceptible (S) populations of *Lolium* species from Chile in comparison to reference’s populations (RP) from Portugal and Spain after the UPGMA analysis performed with AFLP marker data. Twelve plants of each putative population were used for the molecular analysis.

The UPGMA analysis clustered the *Lolium* spp. populations into a large group and two subgroups. In the larger group, the Jaccard index indicated a 62% similarity between *L. rigidum*, *L. perenne*, and *L. multiflorum*. In the first subgroup, the putative R and S *L. rigidum* populations from Chile were confirmed to belong to this species, because they were grouped together to the reference population from Spain. In the second subgroup, *L. multiflorum* and *L. perenne* populations were found to have 80% similarity. The putative R and S populations from Chile of each of these two *Lolium* species were grouped with their respective reference population from Portugal (*L. perenne*) and Spain (*L. multiflorum*), confirming their identities at molecular level (Figure 1).

### Glyphosate Dose–Response Assays

**FIGURE 2:**
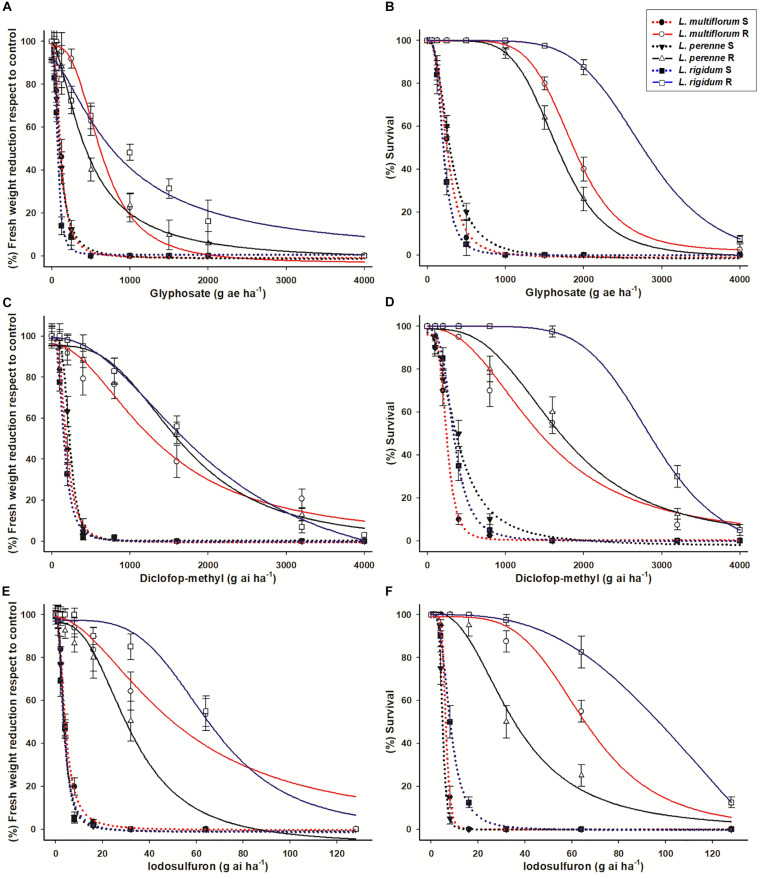
Dose-response curves (fresh weight reduction and survival rates) of glyphosate **(A,B)**, diclofop-methyl **(C,D)** and iodosulfuron **(E,F)** in resistant (R) and susceptible (S) populations of *Lolium* species from Chile. Vertical bars ± standard error (*n* = 8).

The fresh weight of the S *Lolium* spp. populations was reduced by 50% with glyphosate doses close to 100 g ae ha^–1^ (Figures 2A,B). The R populations of *L. perenne*, *L. rigidum*, and *L. multiflorum* were 5- (GR_50_ = 453 g ae ha^–1^), 6.6- (GR_50_ = 689 g ae ha^–1^) and 12.5- (GR_50_ = 937 g ae ha^–1^) times more resistant to glyphosate, respectively, in relation to their respective S counterparts. Regarding survival (LD_50_), the RF values of the R populations of *L. perenne*, *L. rigidum* and *L. multiflorum* were 5.6, 7.1, and 14.1, respectively. These RF values were determined according to the LD_50_ (210, 258 and 290 g ae ha^–1^ glyphosate) values of the S populations following the same species order (Table 2).

**TABLE 2:** Parameters of the sigmoidal equation^a^ used to estimate the effective mean dose of glyphosate (EPSPS inhibitor) required to reduce the fresh weight (GR_50_) and plant survival (LD_50_) by 50% in multiple-resistant (R) and -susceptible (S) populations of *Lolium* species in Chile.

Species	Population	d	b	*R*^2^	Mean dose	RF	*P-*value
**Growth reduction (GR_50_)**							
*L. multiflorum*	R	98.8	2.2	0.99	689.2	6.6	<0.0001
	S	99.5	1.9	0.98	103.7		
*L. perenne*	R	102.4	1.4	0.99	452.9	5.0	<0.0001
	S	102.2	1.8	0.99	90.7		
*L. rigidum*	R	100.2	1.5	0.99	936.6	12.5	<0.0001
	S	100.9	1.8	0.99	74.9		
**Plant survival (LD_50_)**							
*L. multiflorum*	R	99.7	3.5	0.99	1, 846.1	7.1	<0.0001
	S	102.4	1.8	0.99	258.3		
*L. perenne*	R	100.2	3.6	0.99	1, 634.4	5.6	<0.0001
	S	102.6	1.8	0.99	290.0		
*L. rigidum*	R	100.5	2.0	0.99	2, 960.1	14.1	<0.0001
	S	102.8	1.9	0.99	210.2		

*^*a*^*Y* = *d*/1+(*x*/*g*): where *Y* = percentage of fresh weight with respect to the untreated control, *d* = upper limit, *b* = slope of the curve, and *g* = herbicide concentration at the inflection point (i.e., GR_50_ or LD_50_), and *x* = herbicide concentration. Resistance factors (RF = R/S) are the ratio of R to-S GR_50_ or LD_50_. *P* is the level of significance of the non-linear model.*

### Diclofop Dose–Response Assays

Diclofop resistance of the R *Lolium* spp. populations followed the same trend as glyphosate resistance, i.e., the R population of *L. perenne* had the lowest resistance level, followed by *L. multiflorum* (intermediate resistance level), while *L. rigidum* was the most resistant species, both in relation to the percentage of fresh weight reduction as well the plant survival rate by 50% (Figures 2C,D). The GR_50_ values of the S populations ranged from 148 to 254 g ai ha^–1^ diclofop, and the LD_50_ values were between 269 and 388 g ai ha^–1^. The RF of the R populations ranged from 6.4 to 11.4 and from 4.4 to 9.4 in relation to the GR_50_ and LD_50_ values, respectively, of the S populations (Table 3).

**TABLE 3:** Parameters of the sigmoidal equation^a^ used to estimate the effective mean dose of diclofop-methyl (ACCase inhibitor) required to reduce the fresh weight (GR_50_) and plant survival (LD_50_) by 50% in multiple-resistant (R) and -susceptible (S) populations of *Lolium* species in Chile.

Species	Population	d	b	*R*^2^	Mean dose	RF	*P-*value
**Growth reduction (GR_50_)**							
*L. multiflorum*	R	100.5	1.4	0.99	1370.9	7.6	0.0001
	S	97.2	1.3	0.98	179.3		
*L. perenne*	R	99.6	1.7	0.99	1643.7	6.4	0.0001
	S	100.5	1.1	0.99	254.9		
*L. rigidum*	R	99.9	1.7	0.99	1698.7	11.4	0.0001
	S	99.3	1.0	0.99	148.9		
**Plant survival (LD_50_)**							
*L. multiflorum*	R	100.0	1.6	0.99	1476.8	5.5	0.0001
	S	102.9	1.3	0.99	269.7		
*L. perenne*	R	99.8	1.9	0.99	1701.1	4.4	0.0001
	S	100.7	1.5	0.99	388.1		
*L. rigidum*	R	100.5	2.3	0.99	2896.9	9.4	0.0001
	S	101.5	1.6	0.99	309.1		

*^*a*^*Y* = *d*/1+(*x*/*g*): where *Y* = percentage of fresh weight with respect to the untreated control, *d* = upper limit, *b* = slope of the curve, and *g* = herbicide concentration at the inflection point (i.e., GR_50_ or LD_50_), and *x* = herbicide concentration. Resistance factors (RF = R/S) are the ratio of R to-S GR_50_ or LD_50_. *P* is the level of significance of the non-linear model.*

### Iodosulfuron Dose–Response Assays

The R populations of the three *Lolium* species survived iodosulfuron rates higher than the recommended field dose (3.5–5 g ai ha^–1^) (Figures 2E,F). Based on weight reduction, the least resistant species was *L. perenne* (GR_50_ = 27.0 g ai ha^–1^), followed by *L. multiflorum* (GR_50_ = 50.2 g ai ha^–1^). *Lolium rigidum* (GR_50_ = 66.8 g ai ha^–1^) was the most resistant species. The R populations were between 9 and 24 times more resistant to iodosulfuron than the S populations. These levels of resistance decreased in relation to the survival rate, where the LD_50_ values of the S populations ranged from 4.9 to 6.2 g ai ha^–1^ and those of R populations from 36 to 82.8 g ai ha^–1^. The RF of the R populations in relation to the S ones were 7.3, 11.2, and 13.4 for *L. perenne*, *L. multiflorum*, and *L. rigidum*, respectively (Table 4).

**TABLE 4:** Parameters of the sigmoidal equation^a^ used to estimate the effective mean dose of iodosulfuron-methyl-sodium (ALS inhibitor) required to reduce the fresh weight (GR_50_) and plant survival (LD_50_) by 50% in multiple-resistant (R) and -susceptible (S) populations of *Lolium* species in Chile.

Species	Population	d	b	*R*^2^	Mean dose	RF	*P-*value
**Growth reduction (GR_50_)**							
*L. multiflorum*	R	99.8	1.4	0.99	50.2	12.9	<0.0001
	S	100.0	1.9	0.99	3.9		
*L. perenne*	R	102.7	1.0	0.99	27.0	9.0	<0.0001
	S	101.2	1.9	0.99	3.0		
*L. rigidum*	R	100.4	1.2	0.98	66.8	23.9	<0.0001
	S	101.3	1.6	0.99	2.8		
**Plant survival (LD_50_)**							
*L. multiflorum*	R	100.4	2.9	0.99	64.1	11.1	<0.0001
	S	101.5	1.7	0.99	5.8		
*L. perenne*	R	100.4	2.6	0.99	36.0	7.3	<0.0001
	S	101.6	1.7	0.99	4.9		
*L. rigidum*	R	99.5	2.3	0.98	82.8	13.4	<0.0001
	S	99.2	1.7	0.98	6.2		

*^*a*^*Y* = *d*/1+(*x*/*g*): where *Y* = percentage of fresh weight with respect to the untreated control, *d* = upper limit, *b* = slope of the curve, and *g* = herbicide concentration at the inflection point (i.e., GR_50_ or LD_50_), and *x* = herbicide concentration. Resistance factors (RF = R/S) are the ratio of R to-S GR_50_ or LD_50_. *P* is the level of significance of the non-linear model.*

### Enzyme Activity Assays

**FIGURE 3:**
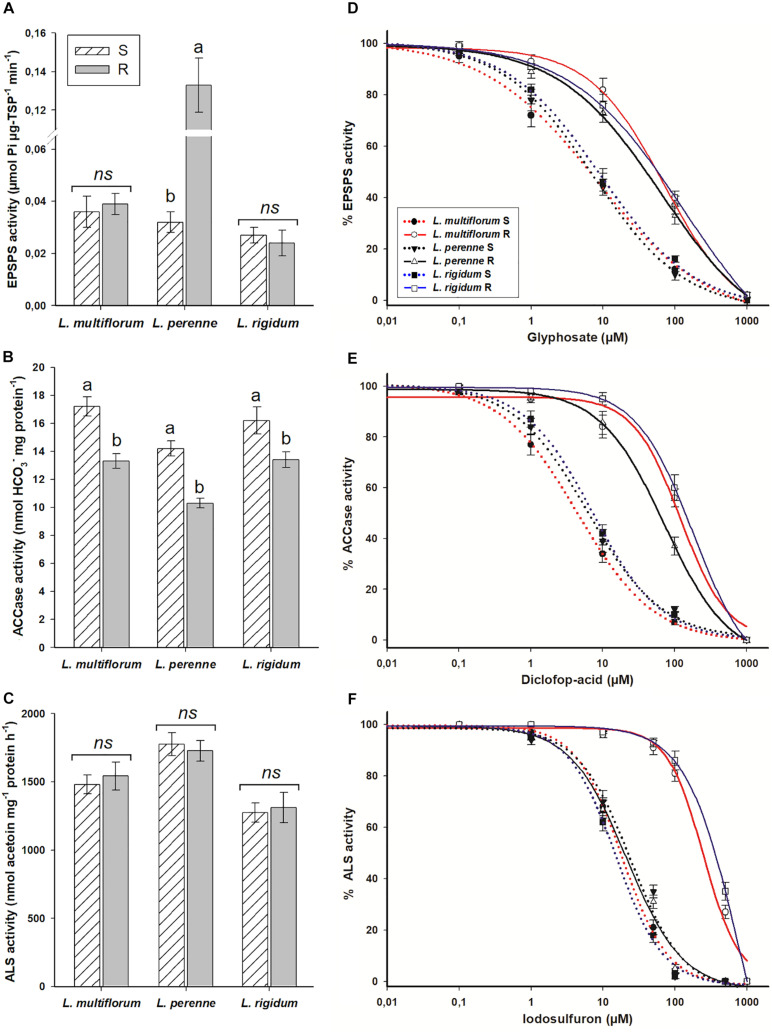
Enzyme activity of resistant (R) and susceptible (S) populations of *Lolium* species in Chile, without and with presence of EPSPS **(A,D)**, ACCase **(B,E)** and ALS **(C,F)** inhibitors. Different letters above bars indicate differences between populations within species according to the Tukey test at 95%. NS, not significant. Vertical bars represent ± SE (*n* = 3).

The specific activity of the EPSPS was similar between R and S populations of *L. multiflorum* (0.037 μmol Pi μg^–1^ TSP min^–1^) and *L. rigidum* (0.026 μmol Pi μg^–1^ TSP min^–1^); however, differences of such activity were observed between *L. perenne* populations [0.032 (S) vs 0.133 (R) μmol Pi μg^–1^ TSP min^–1^]. The specific ACCase activity, that ranged from 10.3 to 17.2 nmol HCO_3_^–^ mg protein^–1^ min^–1^, was lower in the R populations of the three *Lolium* species. In the case of the specific ALS activity, there were differences between species, but not among R and S populations within each *Lolium* species. The averages were 1,511, 1,752, and 1,293 nmol acetoin mg^–1^ protein h^–1^ for *L. multiflorum*, *L. perenne*, and *L. rigidum*, respectively (Figures 3A,C,E).

The R populations of *L. multiflorum*, *L. perenne*, and *L. rigidum* required 14.2, 8.4, and 12.3 times, respectively, more glyphosate to inhibit EPSPS activity by 50%; and 27.7, 11.1, and 29.6 times more diclofop to inhibit ACCase in the same proportion in relation to their respective S counterparts. The ALS activities of the R populations of *L. multiflorum* and *L. rigidum* in response to iodosulfuron were 13.4 and 23.2 times lower, respectively, than the S populations; however, no differences in the sensitivity of this enzyme were observed between the R and S populations of *L. perenne* (Figures 3B,D,F and Table 5).

**TABLE 5:** Parameters of the sigmoidal equation^a^ used to estimate the concentrations (μM) of glyphosate, diclofop-methyl and iodosulfuron-methyl-sodium to inhibit the enzymatic activity of the EPSPS, ACCase and ALS by 50% (I_50_), respectively, in multiple-resistant (R) and -susceptible (S) populations of *Lolium* species in Chile.

Species	Population	D	b	*R*^2^	I_50_	RF	*P-*value
**EPSPS (5-enolpyruvylshikimate-3-phosphate synthase)**							
*L. multiflorum*	R	99.9	14.3	0.98	102.3	14.2	<0.0001
	S	102.2	4.0	0.99	7.2		
*L. perenne*	R	101.6	2.9	0.99	65.3	8.4	<0.0001
	S	102.0	3.1	0.99	7.8		
*L. rigidum*	R	100.3	16.3	0.98	120.7	12.3	<0.0001
	S	100.6	5.2	0.99	9.8		
**ACCase (acetyl-coenzyme A carboxylase)**							
*L. multiflorum*	R	99.4	9.6	0.99	127.6	27.7	<0.0001
	S	101.6	2.0	0.99	4.6		
*L. perenne*	R	99.5	8.9	0.99	68.6	11.1	<0.0001
	S	100.6	2.7	0.98	6.2		
*L. rigidum*	R	99.4	11.5	0.99	186.2	29.6	<0.0001
	S	100.3	2.2	0.99	6.3		
**ALS (acetolactate synthase)**							
*L. multiflorum*	R	100.6	10.5	0.99	245.0	13.4	<0.0001
	S	99.5	6.9	0.99	18.3		
*L. perenne*	R	99.5	10.8	0.99	22.3	0.9	<0.0001
	S	98.3	8.8	0.98	24.0		
*L. rigidum*	R	100.4	8.6	0.99	392.5	23.2	<0.0001
	S	98.7	7.1	0.99	16.9		

*^*a*^*Y* = *d*/1+(*x*/*g*): where *Y* = percentage of enzyme inhibition with respect to the specific activity, *d* = upper limit, *b* = slope of the curve, and *g* = herbicide concentration at the inflection point (i.e., I_50_). Resistance factors (RF = R/S) are the ratio of R-to-S I_50_.*

### Target Site Changes

Nucleotide substitutions were found at the 106-CCA codon, which naturally encodes to proline, in the *EPSPS* gene, in the R *L. multiflorum* and *L. rigidum* populations (Table 6). The most frequent codon substitution was TCA (serine) found in 8 and 12 resistant individuals of *L. multiflorum* and *L. rigidum*, respectively. In addition, five R individuals of *L. multiflorum* presented the ACA (alanine) nucleotide combination. Regarding the *ACCase* gene, the three R populations of *Lolium* species showed different combinations of amino acid substitutions. Four and five R individuals of *L. multiflorum* presented the mutations Ile-2041-Asn and Asp-2078-Gly, respectively; *L. perenne* presented only the Asp-2078-Gly mutation (7 individuals); and *L. rigidum* presented the mutations Ile-1781-Leu and Asp-2078-Gly (5 and 8 individuals, respectively). Finally, the most frequent amino acid substitution found in the *ALS* gene of the R *L. multiflorum* and *L. rigidum* populations was Trp-574-Leu (11 and 6 individuals, respectively). In addition, two different amino acid substitutions were found at the Pro-197 position of the *ALS* gene in four (Ser) and three (Gln) individuals of the R *L. rigidum* population. No nucleotide changes were detected in the R *L. perenne* population (Table 6).

**TABLE 6:** Frequency of amino acid substitutions (% and number of plants in parenthesis) in the genes of the target enzymes 5-enolpyruvylshikimate-3-phosphate synthase (EPSPS), acetyl-coenzyme A carboxylase (ACCase) and acetolactate synthase (ALS) of multiple-resistant (R) *Lolium* spp. populations from Chile.

Gene	Position	Amino acid change	*L. multiflorum*	*L. perenne*	*L. rigidum*
EPSPS	Pro-106	Ser	40 (8)	N.D.	60 (12)
		Ala	25 (5)	N.D.	N.D.
ACCase	Ile-1781	Leu	N.D.	N.D.	25 (5)
	Ile-2041	Asn	20 (4)	N.D.	N.D.
	Asp-2078	Gly	25 (5)	35 (7)	40 (8)
ALS	Pro-197	Ser	N.D.	N.D.	20 (4)
		Gln	N.D.	N.D.	15 (3)
	Trp-574	Leu	55 (11)	N.D.	30 (6)

*Twenty plants were analyzed per population. No mutations were detected in the S populations and therefore, information is not shown. N.D., not detected.*

## Discussion

Differentiation between *Lolium* species on the basis of phenotypic characteristics is difficult (Menegat et al., 2016), therefore, a molecular characterisation was required to establish the molecular identities between the three populations studied. AFLP analyses make it possible to differentiate which populations belong to each *Lolium* species based on their molecular relationships (>90% similarity), by comparing them with their respective reference species of *L. multiflorum*, *L. perenne* and *L. rigidum*. However, this molecular tool did not allow differentiating the susceptibility or resistance status to herbicides since the genetic similarity between R and S populations within each species was equal to or greater than 95%. Due to the lack of homology and size homoplasy of fragments between individuals and populations, AFLP analyses may give biased results (Caballero et al., 2008; Fernández-Moreno et al., 2017a). For example, glyphosate R and S populations of *Amaranthus palmeri* presented high similarity levels of genetic variation (Chandi et al., 2013), as well as populations of *Spartina alterniflora* from different coastal regions of China that showed a weak genetic differentiation (Li et al., 2019). Therefore, AFLPs analyses are important to understand genetic diversity between and within weed populations, but these studies are not so crucial to optimize weed management programs (Sterling et al., 2004).

### Resistance to EPSPS Inhibitor

Among grasses worldwide, *Lolium* species have evolved the greatest number of cases of selection for glyphosate resistance (Heap, 2020). In Chile, the first case of glyphosate resistance was found in *L. multiflorum* in 2001 in fruit orchards in Region VIII (San Bernardo and Olivar) (Perez and Kogan, 2003), where two R populations presented RF 2- to 4-fold higher in relation to a S population. Subsequently, glyphosate resistant populations of the same species were also found in wheat fields of Region IX (Vilcún) with RF of 7.3 (Michitte et al., 2005). Glyphosate resistance levels of the R *L. multiflorum*, *L. perenne*, and *L. rigidum* populations included in this study, which varied between 6- and 11-fold based on growth reduction, and 4- to 9-fold according to plant mortality, were closer to those observed in the R *L. multiflorum* population of Vilcún, as well as to those reported for other glyphosate-resistant populations of *Lolium* spp. (Chandi et al., 2013; Fernández et al., 2017; Fernández-Moreno et al., 2017a,b; Yanniccari et al., 2017).

Herbicide resistance can be conferred by target-site resistance (TSR) and non-target site resistance (NTSR) mechanisms (Gaines et al., 2020). In this study we did not evaluate NTSR mechanisms; however, this does not mean that they cannot be involved in the herbicide resistance observed in the three R *Lolium* populations. Target site mutations at the Pro-106 position of the *EPSPS* gene in *Lolium* species, with Pro substituted by Ser, Thr, or Ala, impart low to intermediate glyphosate resistance levels, with RF ranging from 2 to 15 (Preston et al., 2009). Mutations in this position (Pro-106) narrow the cavity of the glyphosate binding site with EPSPS but maintain the affinity of this enzyme with its substrate (phosphoenolpyruvate), which allows plants exposed to glyphosate field doses to survive (Funke et al., 2006). The mutations found in the *EPSPS* gene of the R populations of *L. multiflorum* (Pro-106-Ser/Ala) and *L. rigidum* (Pro-106-Ser) explain their glyphosate resistance levels, as corroborated in the enzyme activity assays. The occurrence of multiple mutations among R individuals of *L. multiflorum* suggests a complex evolutionary history of the resistance traits, as observed in R *L. multiflorum* populations of northwest California that showed high nucleotide diversity at Pro-106 position between and within populations (Karn and Jasieniuk, 2017). Although *L. perenne* did not present any mutation, its glyphosate resistance could also be explained at target site level, since the R population presented an EPSPS specific activity four times higher than the S population. The high EPSPS specific activity in this R population suggests that there was an overproduction of the EPSPS enzyme, which could be due to increased *EPSPS* gene copy number, or alternatively EPSPS activity could be higher in R populations due to post-translational modifications of the enzyme (Gherekhloo et al., 2017; Gaines et al., 2019). Thus, although the EPSPS of R plants is glyphosate sensitive, higher concentrations of herbicide are required to completely inhibit it (Gaines et al., 2019). *EPSPS* gene amplification was reported as the main TSR mechanism in glyphosate-resistant populations of *L. multiflorum* in the United States (Salas et al., 2012), and *L. perenne* in Argentina (Yanniccari et al., 2017), conferring varying levels of resistance depending on the numbers of copies of the *EPSPS* gene of each population.

### Resistance to ACCase Inhibitors

Resistance to ACCase inhibitors based on *ACCase* gene overexpression is unusual, and it has only been found to confer resistance in *Digitaria sanguinalis* in Canada (Laforest et al., 2017). In our case we can rule out this mechanism, since the ACCase specific activity of the three R *Lolium* spp. populations was slightly lower than in the S populations. This lower specific activity could be due to the Asp-2078-Gly mutation that was found in some individuals of the R populations of *L. multiflorum*, *L. perenne*, and *L. rigidum*. Unlike other mutations, which also confer resistance to ACCase inhibitors such as those found in another R *Lolium* individual (Ile-1781-Leu and Ile-2041-Asn), the Asp-2078-Gly mutation has a fitness penalty for the plants that carry it, which reduces the speed in catalyzing the formation of malonyl-CoA at the expense of natural substrates (acetyl-CoA, ATP, and HCO_3_) (Vila-Aiub et al., 2015). This fitness penalty was observed in *Alopecurus myosuroides* and *L. rigidum* resistant to ACCase-inhibitors, where the specific activity of the ACCase of homozygous R plants of these weeds was reduced by ∼30% in relation to S plants (Vila-Aiub et al., 2015). The mutation Asp-2078-Gly has been reported to confer cross and different levels of ACCase resistance in *L. multiflorum* and *L. rigidum* around the world (Yu et al., 2007). In *L. perenne*, this mutation was found in R populations in Argentina (Yanniccari and Gigón, 2020). The selection of the Asp-2078-Gly mutation in the *ACCase* gene may fully explain the resistance to diclofop in *L. perenne*, and partially those observed in *L. multiflorum* and *L. rigidum* in dose-response and ACCase activity assays, since some individuals presented other mutations that also confer resistance to ACCase inhibitors.

The Ile-1781-Leu and Ile-2041-Asn combinations found in some R individuals of *L. multiflorum* and *L. rigidum*, respectively, contributed to an increase in the resistance to diclofop, but also showed that the R populations of these three *Lolium* species were not homogeneous. As already noted, because *Lolium* species are weeds of obligated cross-pollination, they may carry different alleles for both cross and multiple target-site resistance within populations (Malone et al., 2013; Martins et al., 2014), and in some case, in the same individual (Yu et al., 2007), as observed in clethodim-resistant *L. rigidum* in Australia, where different individuals of nine populations presented multiple ACCase mutations (one population presented 5 mutations), and two ACCase resistant alleles were found in single *L. rigidum* plants of two populations (Saini et al., 2015). The amino acid substitution Ile-1781-Leu is the most common mutation in resistant grass weed species and confers resistance to all classes of ACCase inhibitors (Kaundun, 2014), and does not have a fitness cost (Vila-Aiub et al., 2015). The Ile-2041-Asn mutation may or not confer resistance to ACCase inhibitors, therefore its presence does not imply it. For example, this mutation was reported to confer resistance to clethodim in *L. rigidum* (Yu et al., 2007), and *Phalaris paradoxa* (Hochberg et al., 2009), but not in *A. myosuroides* (Délye et al., 2008). When the Ile-2041-Asn contributes to ACCase resistance, it is responsible for moderate to high resistance levels to ariloxyphenoxypropionates (FOPs) and low to moderate to phenylpyrazolines (DENs) (Ghanizadeh et al., 2019).

### Resistance to ALS Inhibitors

Like for the *ACCase* gene, overexpression of the *ALS* gene is also rare (Zhao et al., 2018). In addition, *ALS* gene overexpression may be insufficient to provide resistance to ALS inhibitors (Wang et al., 2019). Since the specific activity of the ALS was similar between S and R populations within each *Lolium* species, the possible involvement of *ALS* gene overexpression in the resistance to iodosulfuron was ruled out. *ALS* gene sequencing revealed target site mutations in *L. multiflorum* (Trp-574-Leu) and *L. rigidum* (Pro-197-Ser/Gln + Trp-574-Leu). Although multiple mutations that confer resistance to ALS inhibitors in the same individual can be found (Singh et al., 2019), it is important to note that the two mutations found in *L. rigidum* occurred in different individuals. Mutations at Pro-197 (resistance to sulfonylureas) and Trp-574 (cross resistance to imidazolines and sulfonylureas) positions have been found in more than 20 weed species. These are the most common mutations conferring resistance to ALS inhibitors (Moss, 2017), since mutations at these points do not represent a major fitness cost (Yu and Powles, 2014). In *Lolium* species, six different substitutions have been found at these amino acid positions (Pro-197-Ala/Arg/Gln/Leu/Ser/Thr and Trp-574-Leu), conferring moderate to high resistance levels to ALS inhibitors in *L. multiflorum* and *L. rigidum* (Yu et al., 2008; Liu et al., 2014), as well as in others weeds such as *Descurainia sophia* (Deng et al., 2017), and *Rapistrum rugosum* (Ntoanidou et al., 2019).

The Asp-376-Glu and Trp-574-Leu mutations conferred resistance to ALS inhibitors in *L. perenne* (Menegat et al., 2016); however, we found no evidence of their participation in the iodosulfuron resistance of the R *L. perenne* population, which was confirmed by means of the dose-response assays, i.e., TSR mechanisms were not involved in such resistance in this species. Therefore, *L. perenne* resistance to iodosulfuron could presumably be governed by NTSR mechanisms, mainly enhanced herbicide metabolism mediated by major enzyme families of plant-degrading routes such as cytochrome P450, glutathione-*S*-tranferase and/or glycosyltransferase (Jugulam and Shyam, 2019). Considering that the occurrence of both TSR and NTSR mechanisms (mainly herbicide metabolism) in ALS- and ACCase-resistant weeds is widespread (Han et al., 2016; Fang et al., 2019), we cannot rule out the participation of NTSR mechanisms in the resistance of *L. multiflorum* and *L. rigidum.* In addition, some of the mutations found in the ALS and ACCase genes of the R *Lolium* populations from Chile have been reported to confer cross resistance (Murphy and Tranel, 2019). Therefore, future research will be focused in characterizing the NTSR-based iodosulfuron resistance in *L. perenne*, the potential contribution of these mechanisms in the R populations of *L. rigidum* and *L. multiflorum*, as well as possible cross resistance within chemical families of ALS and ACCase inhibitors.

## Conclusion

An accumulation of target site mutations that confer resistance to ALS, ACCase and EPSPS inhibiting herbicides was found in R populations of *L. rigidum* and *L. multiflorum* collected in barley and wheat fields in Chile. The resistance profile to glyphosate, diclofop and iodosulfuron of *L. perenne* differed in relation to the other two species of *Lolium* (annual weeds), possibly due to its perennial habit facilitating vegetative reproduction instead of sexual reproduction. Therefore, in addition to including non-chemical methods in the management of these resistant *Lolium* species, the characterisation of the possible participation of NTSR-based herbicide resistance is essential for the establishment of a properly integrated weed management program. The Chilean farmers should implement crop rotation, the use of mechanical control, sowing variation, and others.

This is the first study in which herbicide resistance mechanisms were characterized in *L. rigidum* from South America. In addition, it is also the first time that multiple resistance to ACCase, ALS and EPSPS inhibiting herbicides have been reported in *L. multiflorum* and *L. perenne* worldwide.

## Data Availability Statement

The raw data supporting the conclusions of this article will be made available by the authors, without undue reservation.

## Author Contributions

JV-G, CP-B, AR-D, HC-H, and RD performed the dose-response and enzyme activity assays. RA, JT, and FB carried out the molecular analysis. RD performed funding project administration and supervision. All authors wrote, corrected and approved the submitted version of this manuscript.

## Conflict of Interest

The authors declare that the research was conducted in the absence of any commercial or financial relationships that could be construed as a potential conflict of interest.
