# Supplementary material for: Accumulation of Target Gene Mutations Confers Multiple Resistance to ALS, ACCase, and EPSPS Inhibitors in Lolium Species in Chile
Source: Front Plant Sci. 2020 Oct 28;11:553948. doi: 10.3389/fpls.2020.553948 (PMC7655540; doi:10.3389/fpls.2020.553948)
Supplement: Supplementary file 1 [file Data_Sheet_1.DOCX]

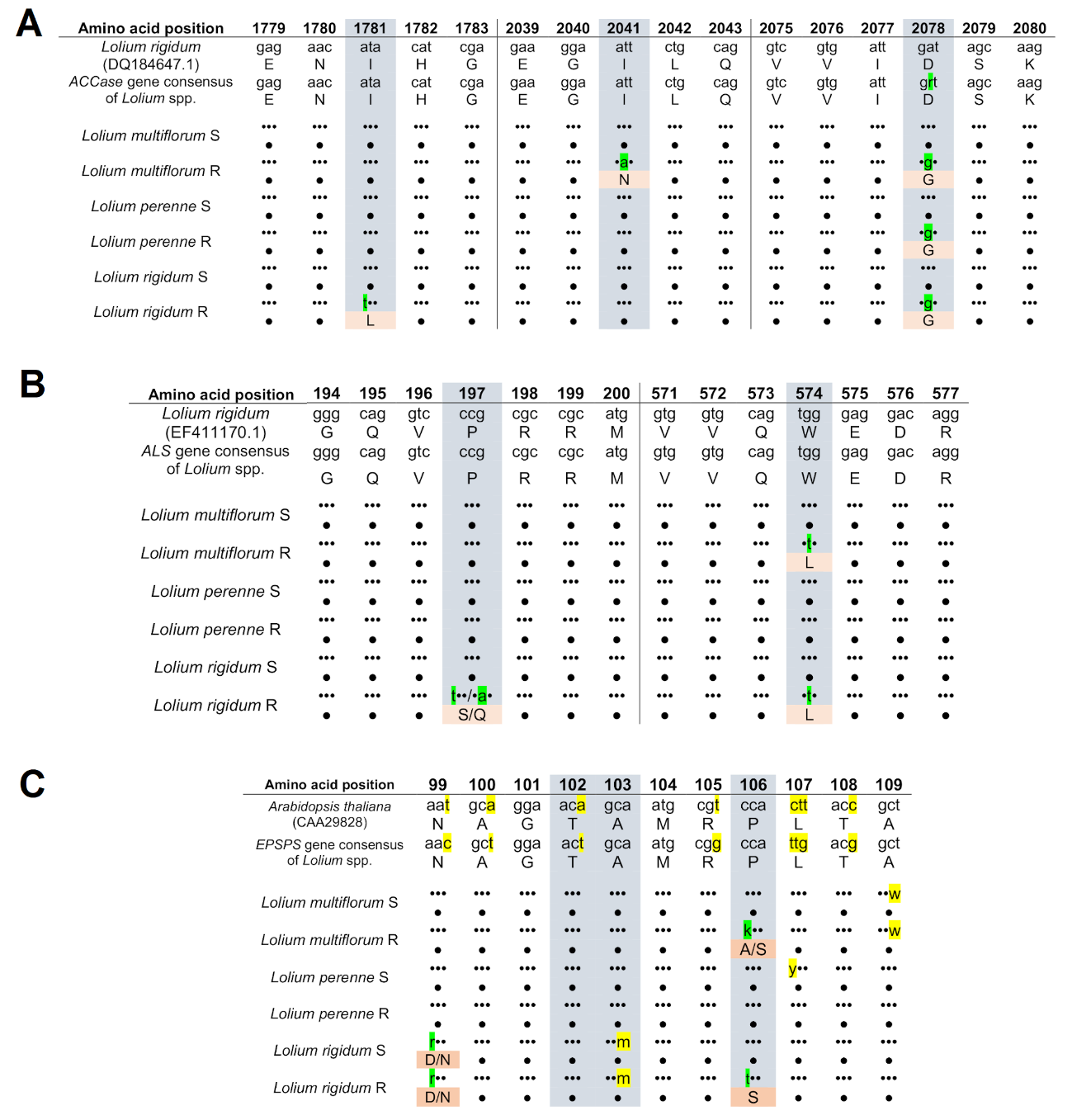


**Figure S1**. Nucleotide sequences of the acetyl CoA carboxylase (ACCase, subfigure A), acetolactate synthase (ALS, subfigure B) and 5-enolpyruvylshikimate-3-phosphate synthase (EPSPS, subfigure C) genes from the multiple herbicide-susceptible and -resistant *Lolium* species from Chile. Blue boxes highlight key positions where an amino acid substitution can confer herbicide resistance. Nucleotides marked in yellow represent single nucleotide polymorphisms (SNPs), in relation to the consensus or reference gene, that do not represent amino acid substitutions. Green SNPs represent amino acid substitution. *k, m, r, w* and *y* indicate ambiguity at a given nucleotide position, i.e., there may be more than one class of nucleotides at that position, depending on the individual or species. k= g or t, m= a or c, r= g or a, y= c or t, and w=a or t.
